# Supplementary material for: Application of human liver organoids as a patient-derived primary model for HBV infection and related hepatocellular carcinoma
Source: eLife. 2021 Jul 30;10:e60747. doi: 10.7554/eLife.60747 (PMC8384419; doi:10.7554/eLife.60747)
Supplement: Transparent reporting form [file elife-60747-transrepform.docx]

***eLife’s* transparent reporting form**

We encourage authors to provide detailed information *within their submission* to facilitate the interpretation and replication of experiments. Authors can upload supporting documentation to indicate the use of appropriate reporting guidelines for health-related research (see [EQUATOR Network](http://www.equator-network.org/%20)), life science research (see the [BioSharing Information Resource](https://biosharing.org/)), or the [ARRIVE guidelines](http://www.plosbiology.org/article/info:doi/10.1371/journal.pbio.1000412) for reporting work involving animal research. Where applicable, authors should refer to any relevant reporting standards documents in this form.

If you have any questions, please consult our Journal Policies and/or contact us: [editorial@elifesciences.org](mailto:editorial@elifesciences.org).

**Sample-size estimation**

- You should state whether an appropriate sample size was computed when the study was being designed
- You should state the statistical method of sample size computation and any required assumptions
- If no explicit power analysis was used, you should describe how you decided what sample (replicate) size (number) to use

Please outline where this information can be found within the submission (e.g., sections or figure legends), or explain why this information doesn’t apply to your submission:

All information regarding the sample size (number of donors, biological replicates and technical replicates), and the relevant statistical methods used are described in the Figure legends and Materials and methods sections. Because of donor variability inherent to the patient-derived or healthy donor human organoid platform, all experiments were performed on at least three independent donors (sample size ≥ 3) and at least three independent times.

**Replicates**

- You should report how often each experiment was performed
- You should include a definition of biological versus technical replication
- The data obtained should be provided and sufficient information should be provided to indicate the number of independent biological and/or technical replicates
- If you encountered any outliers, you should describe how these were handled
- Criteria for exclusion/inclusion of data should be clearly stated
- High-throughput sequence data should be uploaded before submission, with a private link for reviewers provided (these are available from both GEO and ArrayExpress)

Please outline where this information can be found within the submission (e.g., sections or figure legends), or explain why this information doesn’t apply to your submission:

Detailed information pertaining to the number of biological versus technical replicates has been explained in figure legends and methods. Experiments were performed and analyzed using technical replicates at different stages of analysis: for example, triplicate organoid wells were used in drug treatment, infections, transgenic modification, etc), followed by additional technical duplicates (in case of RNA quantitation at both RT as well as PCR stages of the analysis). Exact donor numbers and biological and technical replicates are indicated in Figure legends where relevant.

**Statistical reporting**

- Statistical analysis methods should be described and justified
- Raw data should be presented in figures whenever informative to do so (typically when N per group is less than 10)
- For each experiment, you should identify the statistical tests used, exact values of N, definitions of center, methods of multiple test correction, and dispersion and precision measures (e.g., mean, median, SD, SEM, confidence intervals; and, for the major substantive results, a measure of effect size (e.g., Pearson's r, Cohen's d)
- Report exact p-values wherever possible alongside the summary statistics and 95% confidence intervals. These should be reported for all key questions and not only when the p-value is less than 0.05.

Please outline where this information can be found within the submission (e.g., sections or figure legends), or explain why this information doesn’t apply to your submission:

Detail of statistical analysis for each experiment has been described in the methods section under statistical analysis and also in the legends:

The data were first analyzed by ANOVA, and then each pair was compared through Dunnett's multiple comparisons test (or t-test). A value of p < 0.05 was considered statistically significant. Data are shown as mean ± SD of at least 3 replicate treatments *P < 0.05; **P < 0.01; ***P < 0.001 . The data were analyzed and graphs were depicted by GraphPad Prism software 5 (GraphPad Software, La Jolla, CA, USA). Obtained data from drug screening of infected organoid have 2-8 technical replicates for each donor. Thus, the obtained data from each donor were analyzed by ANOVA separately.

(For large datasets, or papers with a very large number of statistical tests, you may upload a single table file with tests, Ns, etc., with reference to sections in the manuscript.)

**Group allocation**

- Indicate how samples were allocated into experimental groups (in the case of clinical studies, please specify allocation to treatment method); if randomization was used, please also state if restricted randomization was applied
- Indicate if masking was used during group allocation, data collection and/or data analysis

Please outline where this information can be found within the submission (e.g., sections or figure legends), or explain why this information doesn’t apply to your submission:

samples were allocated into experimental groups randomly.

**Additional data files (“source data”)**

- We encourage you to upload relevant additional data files, such as numerical data that are represented as a graph in a figure, or as a summary table
- Where provided, these should be in the most useful format, and they can be uploaded as “Source data” files linked to a main figure or table
- Include model definition files including the full list of parameters used
- Include code used for data analysis (e.g., R, MatLab)
- Avoid stating that data files are “available upon request”

Please indicate the figures or tables for which source data files have been provided:

Figure 1F, Figure 1G, Figure 2B, Figure 2C, Figure 2D, Figure 2F, Figure 3C, Figure 3D, Figure 3E, Figure 1 Supplementary 2C, Figure 1 Supplementary 2D, Figure 4 Supplementary 1B, Figure 4 Supplementary 1C, Figure 5C, Figure 5 Supplementary 1A
